# Sudden activation of temporary cardiac pacing due to postoperative brainstem hematoma in 2 cases

**DOI:** 10.1186/s40981-020-00374-z

**Published:** 2020-09-09

**Authors:** Yuki Terada, Satoki Inoue, Masahiko Kawaguchi

**Affiliations:** Department of Anesthesiology and Division of Intensive Care, Nara Medical University, 840 Shijo-cho Kashihara, Nara, 634-8522 Japan

To the Editor

It has been noted that brainstem lesion surgery especially on the medulla oblongata may cause hemodynamic changes with transient acute bradycardia or asystole and hypotension during surgical procedures [1]. In one such case, temporary cardiac pacing (TCP) served as an effective option for preventing hemodynamic complications induced by surgical procedures [2]. Although TCP during this surgery occasionally activated when the heart rate dropped below the predefined threshold, surgery was not interrupted, and radical tumor removal was successful. We report 2 cases of hemangioblastomas in the cerebellopontine angle (CPA) with intraoperative courses identical to the above-mentioned case report where TCP was periodically activated post-surgery because of hematoma at the surgical lesions.

A 43-year-old man underwent surgical removal of a hemangioblastoma in the CPA and TCP was inserted on the day of surgery after anesthesia induction. The pacemaker was programmed to VVI mode at 50 bpm. During surgical manipulations, the TCP worked frequently and prevented interruption of the procedures. After 8 h of surgery, computed tomography (CT) of the head showed successful decompression but a small remaining tumor lesion. The patient was electively ventilated in the intensive care unit (ICU). One hour after admission to the ICU, the TCP suddenly activated and started pacing at 50 bpm, which had been set as the predefined threshold; the actual heart rate was approximately 20 bpm, which was confirmed by temporarily interrupting the pacing. An urgent head CT showed that a postoperative hematoma compressed the medulla oblongata (Fig. 1a). After surgical removal of the hematoma, the spontaneous heart rate stabilized. The patient remained ventilated for 3 days and then was weaned. The other postoperative course was uneventful although he required a ventriculoperitoneal shunt due to postoperative hydrocephalus. The second case was a 50-year-old man who also underwent surgical removal of hemangioblastoma in the CPA. Fifteen minutes after admission to the ICU, TCP was activated and started back-up pacing of VVI mode at 50 bpm. An urgent head CT showed postoperative hematoma (Fig. 1b) after which he followed a course similar to the previous case.

**Fig. 1:**
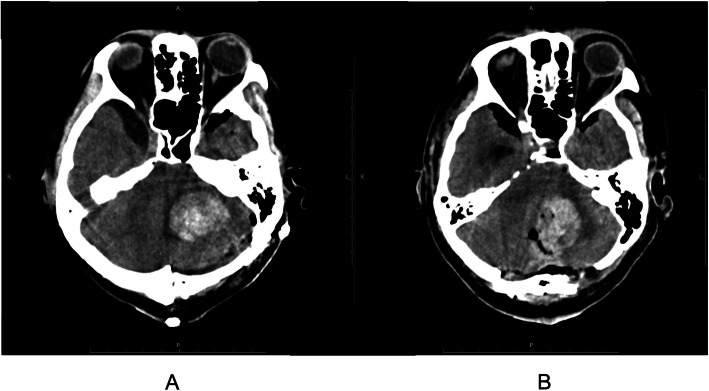
Postoperative intracranial hematoma. Postoperative brain computed tomography showing intracranial hematoma. Hematoma in the left cerebellopontine angle in case 1 (**a**) and case 2 (**b**) was detected by postoperative bran computed tomography examined after the onset of bradycardia in the ICU

From the standpoint of neurosurgical procedures, TCP prevents procedure-induced bradycardia and hypotension and provides a satisfactory condition for tumor removal. Apart from surgical procedures, TCP is an actual lifesaving device for bradycardia. Additionally, TCP may serve as a warning monitor for postoperative hematoma associated with brainstem lesions including those of the medulla oblongata and may facilitate rapid surgical intervention if needed. It is well documented that hemangioblastomas are significantly correlated with an increased risk of a postoperative hematoma [3]. In cases of hemangioblastoma with brainstem lesions such as our 2 cases, TCP may be effective to prevent severe hypo-perfusion during the postoperative period as well as during surgical manipulations.

## Data Availability

Not applicable

## Abbreviations

TCP: Temporary cardiac pacing
CPA: Cerebellopontine angle
CT: Computed tomography
VVI: Ventricle paced, ventricle sensed, and pacing is inhibited if beat is sensed
